# Disruption of Protein Kinase A in Mice Enhances Healthy Aging

**DOI:** 10.1371/journal.pone.0005963

**Published:** 2009-06-18

**Authors:** Linda C. Enns, John F. Morton, Piper R. Treuting, Mary J. Emond, Norman S. Wolf, G. S. McKnight, Peter S. Rabinovitch, Warren C. Ladiges

**Affiliations:** Departments of Comparative Medicine, Biostatistics, Pharmacology, and Pathology, School of Medicine, University of Washington, Seattle, Washington, United States of America; Mayo Clinic College of Medicine, United States of America

## Abstract

Mutations that cause a reduction in protein kinase A (PKA) activity have been shown to extend lifespan in yeast. Loss of function of mammalian RIIβ, a regulatory subunit of PKA expressed in brain and adipose tissue, results in mice that are lean and insulin sensitive. It was therefore hypothesized that RIIB null (RIIβ^−/−^) mice would express anti-aging phenotypes. We conducted lifespan studies using 40 mutant and 40 wild type (WT) littermates of equal gender numbers and found that both the median and maximum lifespans were significantly increased in mutant males compared to WT littermates. The median lifespan was increased from 884 days to 1005 days (p = 0.006 as determined by the log rank test) and the 80% lifespan (defined here as 80% deaths) was increased from 941 days to 1073 days (p = 0.004 as determined by the Wang-Allison test). There was no difference in either median or 80% lifespan in female genotypes. WT mice of both genders became increasingly obese with age, while mutant mice maintained their lean phenotype into old age. Adiposity was found to correlate with lifespan for males only. 50% of male mice between 30 and 35 g, corresponding to about 5% body fat, for either genotype lived over 1000 days. No male mouse outside of this weight range achieved this lifespan. During their last month of life, WT mice began losing weight (a total of 8% and 15% of body weight was lost for males and females, respectively), but RIIβ^−/−^ male mice maintained their lean body mass to end of life. This attenuation of decline was not seen in female mutant mice. Old male mutant mice were insulin sensitive throughout their life. Both genders showed modestly lower blood glucose levels in old mutants compared to WT. Male mutants were also resistant to age-induced fatty liver. Pathological assessment of tissues from end of life male mutant mice showed a decrease in tumor incidence, decreased severity of renal lesions, and a trend towards a decrease in age-related cardiac pathology. These findings help establish the highly conserved nature of PKA and suggest that disruption of PKA affects physiological mechanisms known to be associated with healthy aging.

## Introduction

cAMP-dependent protein kinase A (PKA) is the best characterized kinase member of the protein kinase superfamily. It mediates signal transduction and transcription for many cellular processes, and plays a major role in the control of triglyceride storage and metabolism in response to nutrient status. PKA activity is triggered by G protein-coupled receptors in response to numerous neurotransmitters and hormones, and is mediated by increasing intracellular cAMP levels through the modulation of adenylyl cyclase (AC) activity [1]. Studies in yeast have shown the AC/PKA pathway to be of importance to longevity. Loss of function of CYR1, an AC ortholog, extends lifespan [2]. Reducing activity of the yeast adenylate cyclase CDC35, the GTP-GDP exchange factor CDC25, or the PKA catalytic subunits TPK1, TPK2 and TPK3, results in lengthened life span [3]. Inhibition of the cAMP-PKA pathway in yeast genetically mimics caloric restriction (CR), the only non-genetic intervention known to increase lifespan in a wide range of organisms from yeast to mice [3]. The effect of down regulation of the AC/PKA pathway on aging and age-related disease in mammals has not been extensively studied [4]. In this regard, it is of interest that disruption of the AC isoform type 5 adenylyl cyclase (AC5) in mice was reported to increase lifespan and resistance to cardiac stress in mice [5]. However, little is known about the age-related effects of deleting specific PKA subunit genes.

Mammalian PKA consists of two regulatory and two catalytic subunits [1]. In the mouse, there are four regulatory and two catalytic isoforms, each encoded by a separate gene [6]. RIIβ (MGI:97760) is one of the regulatory isoforms and is predominantly expressed in brown and white adipose tissue and in brain [1], [7], tissues known to be important in the regulation of energy homeostasis. Nearly all PKA activity in adipose tissue and 50% of PKA activity in the striatum, hypothalamus, and cortex is attributed to the RIIβ subunit [7]. Partial compensation for the loss of RIIβ occurs in these tissues with up-regulation of the RI regulatory subunit [8]–[10]. RIIβ null (RIIβ^−/−^) mice are lean in comparison to their wild type littermates, have increased resting metabolic activity, body temperature, uncoupling protein 1 (UCP1) concentrations, and lipid hydrolysis [7]. Loss of the RIIβ subunit has been shown to protect mice on a high-fat, high-carbohydrate diet against weight gain, hyperinsulinemia, fatty livers and insulin resistance, reduce plasma levels of VLDL and LDL cholesterol, and improve glucose dispersal [6]. Lower serum glucose and cholesterol levels and improved insulin sensitivity are known to occur during caloric restriction [11], [12].

We therefore hypothesized that old RIIβ^−/−^ mice would be characterized by anti-aging phenotypes based on data showing that disruption of PKA extends lifespan in yeast, as well as the anti-obesity and anti-diabetic phenotypes of these mice. Our preliminary findings help establish the highly conserved nature of PKA and suggest that disruption of PKA affects physiological mechanisms known to be associated with healthy aging.

## Results

### PKA RIIβ null mice are long-lived

**Figure 1:**
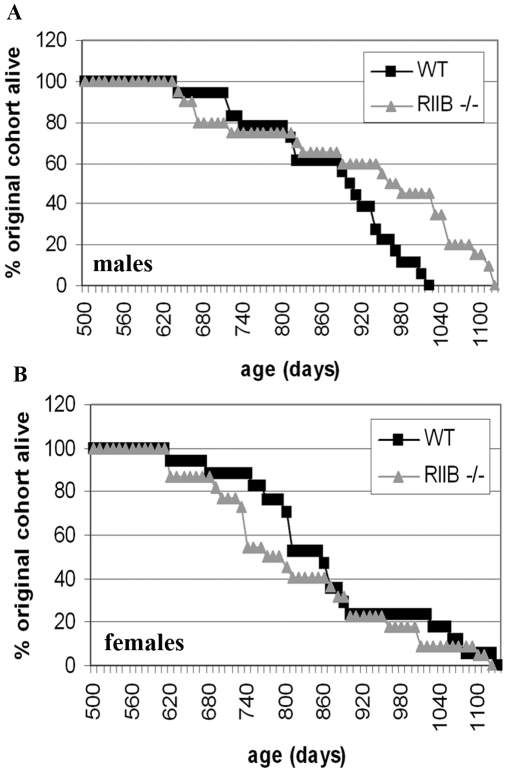
A. Disruption of the RIIβ subunit of Protein Kinase A increases lifespan in male mice on the C57BL/6 background. A. Both the median and maximum lifespans were significantly increased in mutant males compared to WT littermates. The median lifespan was increased from 884 days to 1005 days (p = 0.006 as determined by the log rank test) and the maximum lifespan (defined here as 80% deaths) was increased from 941 days to 1073 days (p = 0.004 as determined by the Wang-Allison test). B. There was no difference in either median or maximum lifespan in female genotypes.

We show that both the median and 80% lifespans (age at which 80% of cohort is dead) are significantly increased in PKA RIIβ mutant males (Figure 1A) (P = 0.006 for the log rank median lifespan test and p = 0.004 for the Wang-Allison 80% lifespan test) but not RIIβ mutant females (Figure 1B). Note that the P-values take into account the small sample size; e.g. there is only a 0.6% chance that this difference would have been seen in the experiment even with only 20 mice if there is no underlying difference. Also note that the difference in male mice is significant even after adjustment for multiple testing of all mice and then the sex subsets (P = 0.02 and P = 0.012, respectively). Therefore, these mice fulfill the prediction for delayed aging based on extended lifespans in yeast with loss of function of PKA gene homologs.

### Adiposity correlates with lifespan for male mice

**Figure 2:**
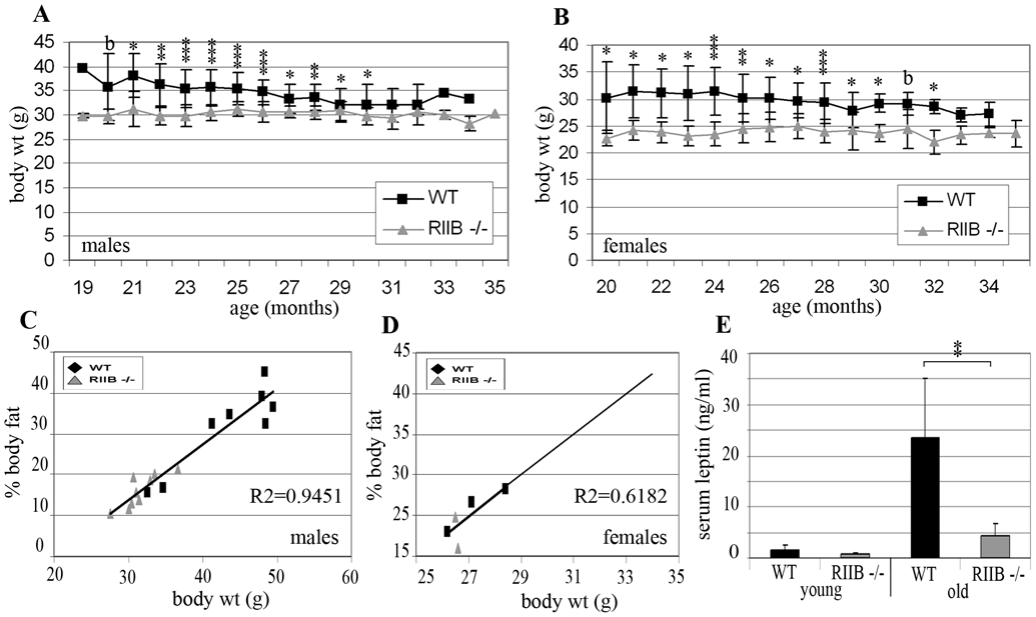
RIIβ^−/−^ mice are resistant to age-related obesity. A,B. RIIβ^−/−^ mice maintain lower body weights than WT littermates. Body weights for RIIβ^−/−^ and WT littermates were measured weekly beginning at 19 to 24 months of age. Measurements were started with 15 mice per gender per genotype. Measurements taken in the last month of each individual mouse's life were not included. Each point represents a mean. Error bars represent standard deviations. N≤15 (sample size decreased with time). b = borderline significance; *P<0.05; **P<0.001; ***P<0.0001 (as determined by Student's t-test). C,D. Body weight and body fat are directly proportional. Body weight and percentage body fat were measured in both old (18 month old) males and females (8 WT and 9 RIIβ^−/−^ males; 3 heterozygous RIIβ^+/−^ and 2 RIIβ^−/−^ females) and found to be directly proportional for both genders (R^2^ = 0.9774 and 0.6182, respectively). E. Old RIIβ^−/−^ mice are resistant to age-induced increases in serum leptin. Serum leptin for young (2 month) and old (16 month) male, RIIB^−/−^ and WT littermates (5 WT and 4 RIIβ^−/−^; and 8 WT and 9 RIIβ^−/−^, respectively). Data presents as means. Error bars represent standard deviations. **P<0.001.

At 2 to 5 months of age, both male and female mutants had body weights approximately 10% less than those of WT littermates (data not shown). By 19 to 20 months of age, both male and female WT littermates were approximately 25% heavier than the respective RIIβ mutant mice (Figure 2A,B). When old (18 month) mice were examined, it was found that for both genders and genotypes, body weight was directly proportional to percentage body fat (Figure 2C,D). Therefore, RIIβ mutants are resistant to age-related obesity. It was also found that 16 month-old WT, male mice incurred 10-fold higher levels of serum leptin than young (2 month-old) WT mice, while RIIβ^−/−^ littermates maintained low leptin levels with age (Figure 2E). Serum leptin levels in heterozygous RIIβ^+/−^ females did not appear to rise as dramatically with age and therefore attenuation in the mutants was not as marked (data not shown), although there was not a sufficient sample size for statistical analysis.

**Figure 3:**
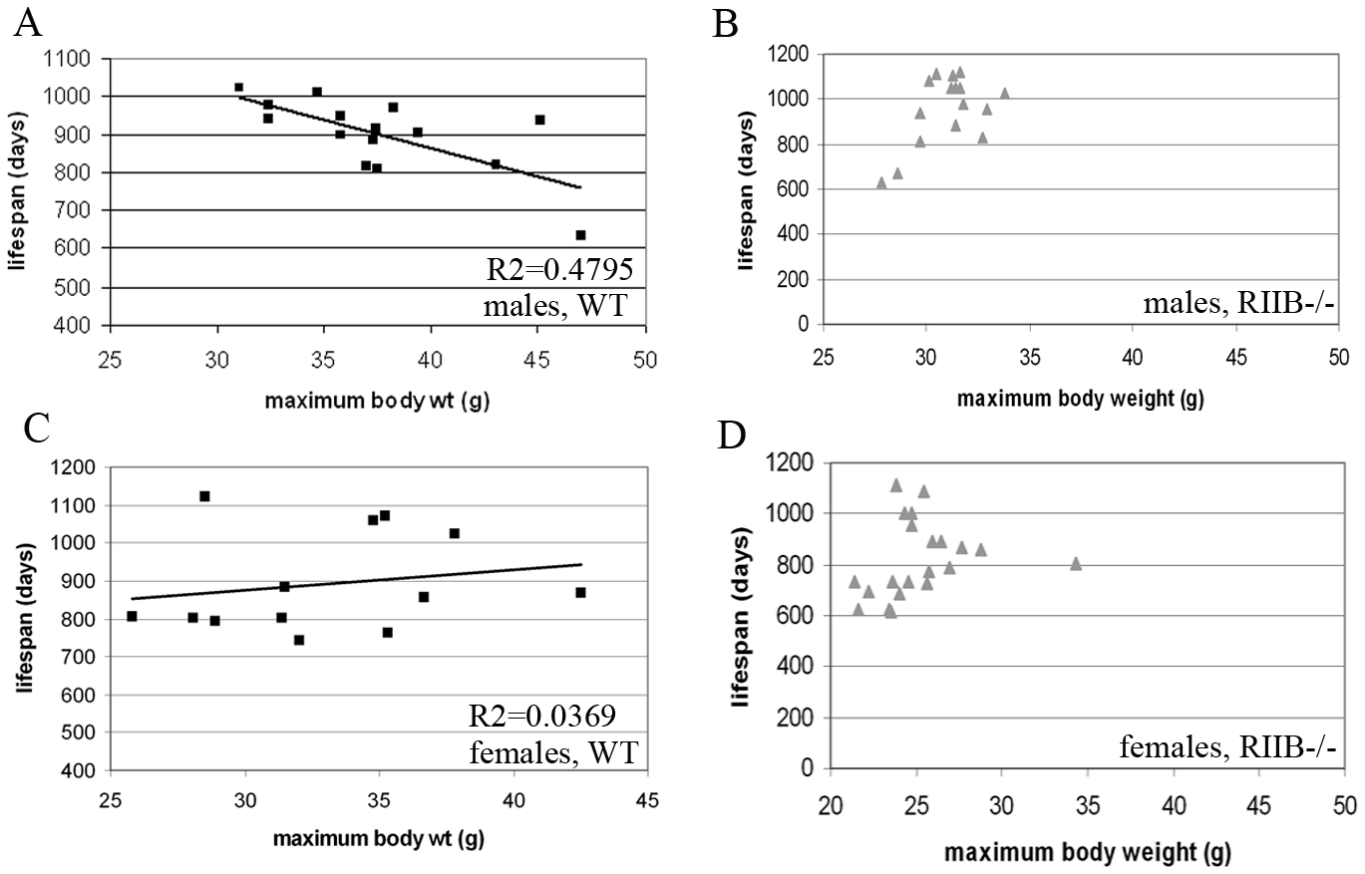
Adiposity correlates with lifespan for males only. A, C. When lifespan of WT mice was plotted against their maximal body weight, a correlation was found for males, but not females (N = 13 and 15, and R^2^ = 0.4795 and 0.0369, respectively). Males with the longest lifespans (over 1000 days) had body weights between 30 and 35 g. B. Most (82%) of the RIIβ^−/−^ male mice examined had body weights between 30 and 35 g. 50% of these mice lived over 1000 days, and the 3 mice that fell outside of this weight range had shortened lifespans of under 700 days (Total N = 17). D. Of the 20 female RIIβ^−/−^ mice measured, only 4 lived to 1000 days or higher. All 5 mice with maximal body weights below 24 g had shortened lifespans equal to or under 800 days, as did the one mouse above 35 g, but lifespans for mice falling between these two body weights were variable.

Body weight, which we found to correspond directly to % body fat, correlated with lifespan in WT mice for males, but not females (Figure 3A,C). Most RIIB^−/−^ male mice had maximal body weights between 30 and 35 g (Figure 3B). About half of the mutants within this weight range lived over 1000 days. The two mutants below 30 g had shortened lifespans of less than 700 days, as did the one mutant that had a maximal body weight above 35 g. Of the male, WT cohort, only 4 mice had maximal body weights within this weight range, and 2 of them lived beyond 1000 days - the longest lifespans in this cohort. Female RIIβ^−/−^ mice all had maximal body weights between 20 and 30 g, and did not appear to have an optimal body weight for extended lifespan (Figure 3D).

### Absence of PKA RIIβ protects mice from fatty liver, insulin resistance, age-related hyperinsulinemia, and cardiac dysfunction

**Figure 4:**
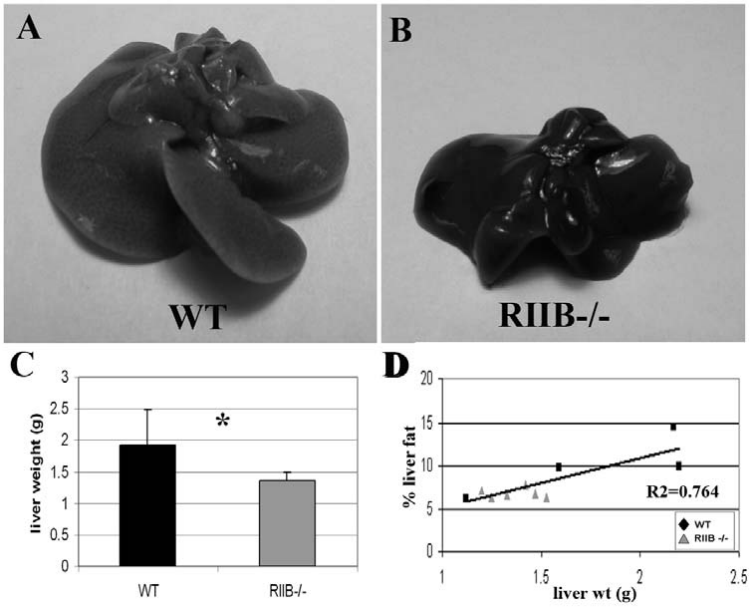
Old RIIβ^−/−^ male mice have attenuated age-related fatty livers. A. Old (18 month old) WT mice developed livers that were twice the size of, and paler in color than livers from young mice. B,C. Livers from 18 month-old RIIβ^−/−^ littermates were smaller, darker in color, and 25% lower in weight than those from their WT littermates. N = 5 (WT) and 6 (RIIβ^−/−^). D. Liver weight correlated directly with % fat content (R^2^ = 0.7064) N = 4 (WT) and 6 (RIIβ^−/−^). For both C and D: Numbers represent means. Error bars represent standard deviations. P's determined by Student's t-test.

Livers were dissected out of 18 month-old, male mice (5 WT and 6 RIIβ^−/−^). It was found that with age, WT mice developed livers up to twice the size of a younger WT liver (Figure 4A). Livers of RIIβ^−/−^ mice weighed on average approximately 25% less than those of WT mice and were darker in color (Figure 4B,C). QNMR analysis revealed a direct correlation between the weight of the liver and its % fat content (Figure 4D).

**Figure 5:**
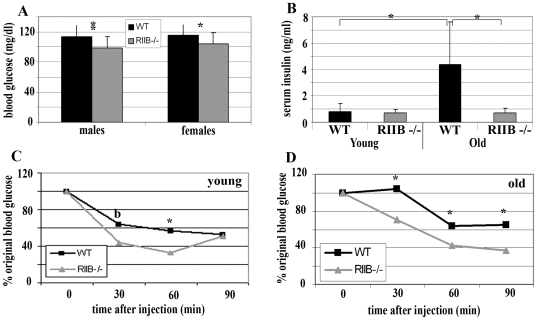
Both young and old RIIβ^−/−^ male mice are insulin sensitive compared to WT, and RIIβ^−/−^ males are resistant to age-induced hyperinsulinemia. A. RIIβ^−/−^ blood glucose levels were slightly lower than WT for both old males and females, but both genotypes of both genders fell into an acceptable range. N = 15. B. Male WT mice experienced over 4-fold increases in serum insulin levels with age, while *RIIβ^−/−^* littermates maintained low serum insulin levels between 2 and 16 months of age. 2 month-old mice: N = 5 (WT) and 4 (RIIβ^−/−^). 16 month-old mice: N = 8 (WT) and 9 (RIIβ^−/−^). C,D. Blood glucose levels of both 2 to 5 month (C) and 18 month (D) RIIβ^−/−^ males dropped more rapidly and further in response to an inter-peritoneal insulin injection. N = 6; points represent means. Results were standardized by setting initial blood glucose levels at 100%. Data for all figures: Numbers represent means, and error bars represent standard deviations. **P<0.001; *P<0.05 and b represents borderline significance (as determined by Student's t-test).

Blood glucose levels for 24-month-old PKA RIIβ mutant males and females were significantly lower than respective WT littermates (p≤0.01) (Figure 5A). However, levels for genotypes of both sexes fell within an acceptable normal range between 100 and 115 mg/dl. Therefore, blood glucose levels are not likely directly associated with extended lifespan. Serum insulin levels for male, WT mice increased 4-fold with age, while RIIB^−/−^ littermates maintained low serum insulin levels between 2 and 16 months of age (Figure 5B). Serum insulin levels in heterozygous RIIβ^+/−^ females did not appear to rise as dramatically with age and therefore attenuation in the mutants was not as marked (data not shown). We injected insulin into a separate cohort of young (2 to 5 month) and old (18 month) RIIβ mutant males and WT littermates. Both age groups of mutant mice were more insulin sensitive than WT littermates (p≤0.05) (Figures 5C and D) suggesting an association with lower blood glucose levels in males. Young (2 to 5 month) females were also tested, and no differences were found between genotypes (data not shown).

**Figure 6:**
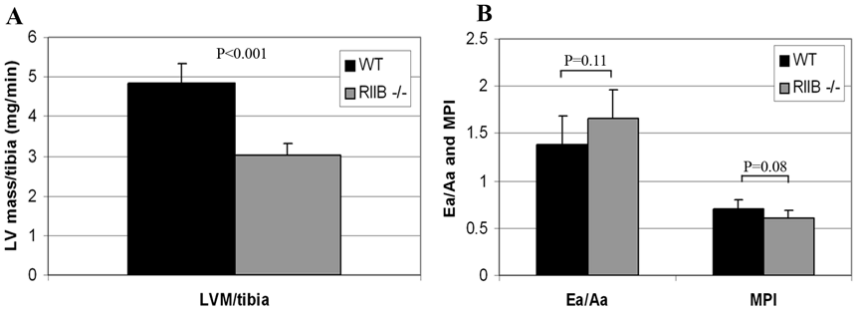
Old RIIβ^−/−^ male mice have attenuated cardiac dysfunction. A. RIIβ^−/−^ mice were significantly protected from age-related LV hypertrophy, shown by lower LV mass normalized to tibia length in 24 month old WT and RIIβ^−/−^ mice. B. These RIIβ^−/−^ mice also had superior Ea/Aa ratios and myocardial performance indices (MPI) compared to WT littermates. N = 6. Numbers represent means. Error bars represent standard deviations. P's determined by Student's t-test.

We chose to use echocardiography to evaluate cardiac function because we can see evidence of dysfunction as early as 15 months of age in C57BL/6 wild type mice, which continually progresses with increasing age (Dai et al., 2009). In a cross-sectional cohort of six male mice per genotype at 24 months of age, RIIβ mutant mice were significantly protected from age-related left ventricular (LV) hypertrophy compared with WT littermates (p<0.001, Fig. 6A). Because of the size differences, we standardized all measurements to tibial length. RIIβ^−/−^ mice also showed a trend toward better both Ea/Aa ratios and myocardial performance indices compared to WT littermates (6B).

### PKA *RIIβ* ^−/−^ females but not males lose weight during old age

**Figure 7:**
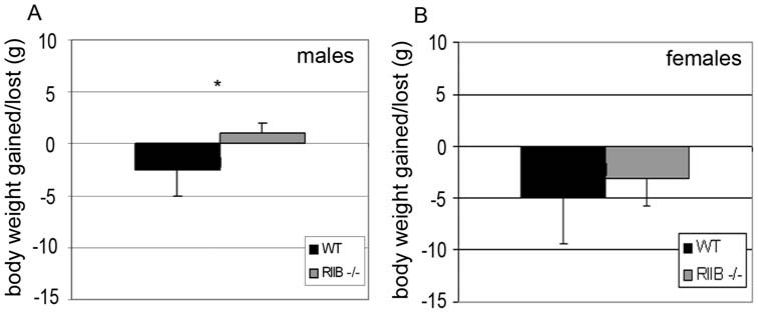
RIIB^−/−^ male mice do not lose weight during aging. Weights of individuals were compared between the first body weight measurements at 19 to 24 months of age and the final measurements taken during the last month of life. WT mice showed an average loss of several grams during senescence (A,B) while male RIIβ^−/−^ littermates maintained their body weight (A). This phenomenon was not seen in RIIβ^−/−^ females (B). Data presented as means. Error bars represent standard deviations. N = 15. *P<0.05 (as determined by Student's t-test).

When weights of individual mice at 19 to 20 months of age were compared to measurements taken during the last month of life, WT males showed an average loss of several grams, while RIIβ mutant littermates maintained their weight (p≤0.05) (Figure 7A). This phenomenon was not observed in females, for which both genotypes lost weight during the measurement period (Figure 4B). This observation may be directly related to extended lifespan in males but not females, or may be an indirect consequence of other age-related conditions in these mice. Both genotypes eat about the same amount, so the body composition differences cannot be attributed to differences in food intake.

### Absence of PKA RIIβ protects mice from age-related pathology

**Figure 8:**
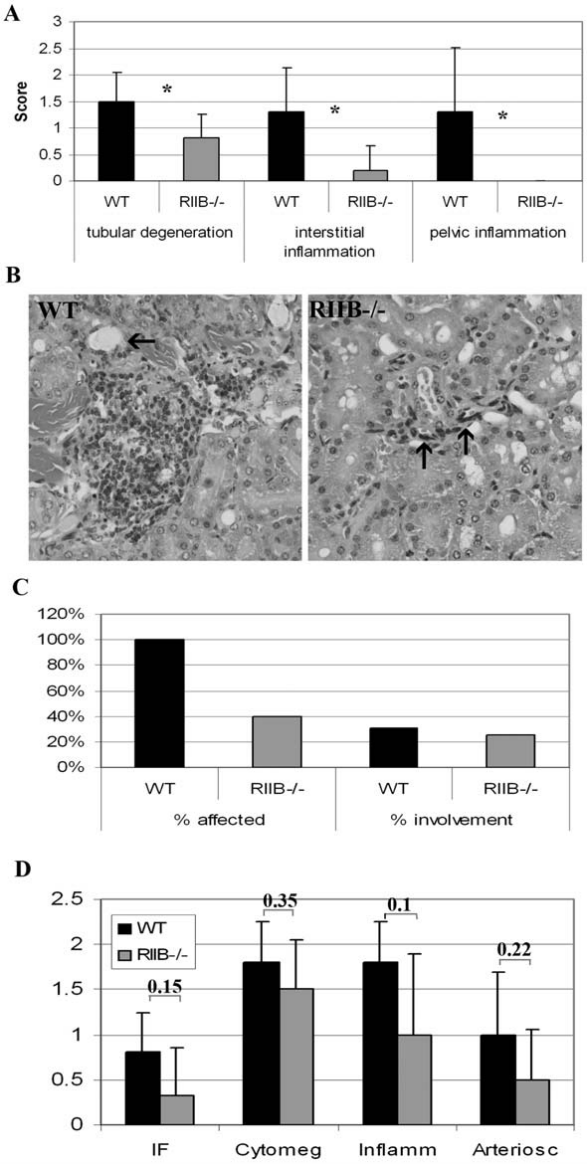
Old RIIβ^−/−^ male mice show attenuated renal dysfunction, tumor incidence, and a trend towards attenuated cardiac pathology. A. Old RIIβ^−/−^ male mice showed attenuated tubular degeneration and interstitial and pelvic inflammation compared to wild-type littermates although renal lesions in both genotypes was relatively mild. N = 6. Numbers represent means. Error bars represent standard deviations. *P<0.05 (determined by Student's t-test). B. Hematoxylin and eosin-stained section of kidneys from WT and RIIβ^−/−^ male mice. The WT mouse had mild perivascular and interstitial lymphohistiocytic accumulations (severity score 2) and mild tubular changes including attenuated epithelium and intralumenal proteinaceous material (arrow). This mouse also had marked neutrophilic pyleitis that likely contributed to moribundity. In contrast, the RIIβ^−/−^ mouse had minimal interstitial inflammation (severity score 1) surrounding a small caliper tangentially sectioned vessel (arrows). Original magnification for both images, 20X. C. Spleens from 100% of WT mice but only 40% of RIIβ^−/−^ mice examined at end of life, presented with lesions. N = 6. D. Hearts from old (18 month) RIIβ^−/−^ male mice tended towards better scores than WT for 4 of 6 types of lesions graded, although differences were not significant. IR = interstitial fibrosis, cytomeg = cytomegaly, inflamm = inflammation, arteriosc = arteriosclerosis. N = 5 (WT) and 6 (RIIβ^−/−^). Numbers represent means. Error bars represent standard deviations. Numbers above columns represent probability values, determined by Student's t-test.

Pathological evaluation was limited because tissues from a number of mice found dead were not suitable for histological fixation. This did provide increased confidence that the end of life end point had been attained, but the relatively low starting cohort numbers (20 per genotype per gender) precluded extensive histopathological assessment. Tissues, collected from old mice that were euthanized because they were determined to die within several hours, were immediately fixed in 10% buffered formalin, stained with hematoxylin/eosin and read by a board certified veterinary pathologist with expertise in mouse pathology (P. Treuting). Evaluations were done on tissues from 6 mutant and 6 WT males at end of life. We were able to determine significant differences between genotypes in renal scores for 3 of 10 different types of graded renal lesions. Tubular degeneration and both interstitial and pelvic inflammation showed significantly better scores in RIIβ^−/−^ males compared to WT (Figure 8A,B). Liver and spleen were examined for tumor incidence and % involvement. No tumor differences were seen in liver (data not shown), but tumors in the spleen were seen in all of the WT mice and in only about 40% of mutant mice (Figure 8C). Heart tissue from 18 month-old mice (5 WT and 6 RIIβ^−/−^) was also examined. While different types of heart lesions from both genotypes were mild on average, it was found that there was a trend for the mutants to have better scores. The differences, however, were not significant (Figure 8D). Looking at hearts from higher numbers of, or of older mice with more severe lesions, might yield more significant differences. These observations suggest that absence of PKA RIIβ suppresses tumor incidence and other age-related pathology.

## Discussion

We have shown that disruption of PKA RIIβ in mice extends lifespan and protects against age-related obesity, weight loss at end of life, cardiac hypertrophy, incidence and severity of age-related pathology, and insulin resistance. Although the mechanisms for the extended lifespan are not yet known, the fact that it is observed only in males provides a uinique paradigm for further investigation of this interesting gene pathway. In this regard, we show preliminary evidence to suggest that the lifespan phenotype seen in the absence of RIIβ is directly associated with decreased body fat but not insulin sensitivity. We also show that RIIβ null males have less age-related fatty liver, cardiac hypertrophy, cancer and renal pathology.

During maturity, many of the WT mice of both genders showed a gradual but significant increase in body weight, found to be associated with an increase in fat mass. Long-lived RIIβ^−/−^ males weighed less, and had less body fat than their WT littermates. In contrast, there was no correlation between adiposity and lifespan in *RIIβ^−/−^* females. Gender-related phenotypic differences have been found in other long-lived mouse models such as the Ames dwarf mouse which shows a greater lifespan increase in females [13]. Sexual dimorphism of lifespan extension is also seen in flies [14], [15]. Such differences are believed to be the consequence of interplay between the mutated pathways and hormones or other growth factors which have sex-specific secretion patterns [16], [17]. There are sex differences in the incidence and presentation of most diseases [18]. In mice, sex differences are mediated by sex chromosome-encoded proteins as well as gonadal steroids [19]. The majority of sexually dimorphic traits are the result of sex differences in expression of the same gene [20], thousands of which, in adipose tissue, show sexual dimorphism [21]. A study by Nas et al., 2009 [22], looked for sex-specific gene coexpression networks in mice and found strong body fat and lipid correlations with a sex-specific module in adipose tissue. This is not surprising, since adipose tissue is an endocrine-responsive organ that interacts with sex hormones [22]. Interestingly, genes in this module are known to be involved in metabolism and obesity, and include leptin, PPAR-α, and genes involved in the inflammatory response [22]. As WT mice age and accumulate increased fat stores, differences in expression of these genes between genders may explain differences in both the susceptibility of males and females to different diseases, as well as to their phenotypic response to a lack of the RIIβ subunit.

Another factor which may contribute to the observed gender specificity of the longevity phenotype is differences in fat distribution between genders. Less important to health than overall adiposity is the type of fat. Sex differences in body fat distribution are well recognized, with males storing more fat in the visceral area than females [23]. Increases in visceral adiposity are a common feature of aging, and in humans are associated with both diabetes and mortality from cardiovascular disease [24]–[28]. Removal of the visceral fat pad from old, insulin resistant rats restores their peripheral and hepatic insulin action to the levels of young rats, and decreases the expression of tumor necrosis factor-α and leptin in subcutaneous adipose tissue [29]. While our study showed increased adiposity in both old male and female WT mice, and attenuation by the RIIβ mutation in both genders, we only looked at overall adiposity. Fat distribution in RIIβ^−/−^ mutants remains to be investigated.

Not surprisingly, given their reduced adiposity, old RIIβ^−/−^ mice were found to have lower serum levels of leptin than WT mice. In humans, plasma leptin levels, which increase with obesity, are a predictor of heart disease, stroke and cardiovascular mortality in males only [30]–[35]. In mice, studies looking at the role of lifespan regulation are contradictory. Harper et al., 2003 [36], observed a negative effect of high levels of serum leptin on lifespan. However, in the long-lived fat cell-specific insulin receptor knockout mouse, lifespan extension occurs in the presence of an increase in plasma leptin. These mice still have a reduced fat mass, as well as low levels of insulin, and this data has been interpreted to mean that insulin levels are a more critical factor in prolonging life in mammals than leptin [37], [38]. It is unknown at this point whether the lower serum leptin levels in RIIβ^−/−^ mice play a role in their longevity, or are merely an indirect result of their reduced adiposity.

RIIβ^−/−^ males showed a decrease in liver fat that paralleled body weight and hence also correlated with lifespan. Hepatic adiposity is related to obesity. Male RIIβ^−/−^ mice were found to be resistant to age-associated fatty livers. Non-alcoholic fatty liver disease (NAFLD) encompasses a spectrum of liver disease from steatosis to cirrhosis and is defined by the American Association for the Study of Liver Diseases as fat accumulation in the liver exceeding 5% to 10% by weight [39]. Fatty livers are related to obesity, perhaps due to adipokines released from adipose tissue, or to exposure of the liver to free fatty acids released from adominal adipose tissue [40]. We found that the fat content of livers of our male mice was directly proportional to their % body fat. It has also been found that parental longevity may be associated with decreased NAFLD in humans, and that this association is even stronger in men [41]. The increased association of fatty liver with lifespan in males points to protection from fatty liver as a possible physiological mechanism for the extended lifespan seen in RIIβ^−/−^ males.

Studies on different long-lived mouse models suggest that the role of insulin in longevity is complex. Mutants such as the Ames and Snell dwarf mice show both extended lifespan as well as increased insulin sensitivity [42], [43]. However, several reports have shown increased lifespan in relatively insulin resistant mouse lines such as IRS-1 and IRS-2 [44], [45], while the fat specific insulin receptor knock out mouse (FIRKO) has reduced insulin-stimulated glucose uptake in adipose tissue but increased systemic insulin sensitivity [37], [38]. Male RIIβ^−/−^ mice were found to be insulin sensitive compared to WT mice, regardless of age. It is, however, unlikely that the increase in lifespan of male RIIβ^−/−^ mice bears any relation to their increased insulin sensitivity, as we did not find differences in insulin sensitivity between young and old WT mice, and the modestly lower blood glucose levels found in the mutants did not show gender specificity. Therefore, while RIIβ^−/−^ mice may have enjoyed health benefits due to chronic insulin sensitivity over the course of their lifespan, insulin sensitivity in old RIIβ^−/−^ mice did not reflect attenuation of an age-related dysfunction, and the insulin sensitivity seen in the male mutants is probably not the cause of their extended lifespan. Interestingly, while the insulin resistance test did not show significant differences between young and old either WT or RIIβ^−/−^ mice, and while neither genotype became hyperglycemic with age, old WT mice did develop hyperinsulinemia. While the attenuation of age-induced obesity in RIIβ^−/−^ mutants does not seem to promote longevity and healthful aging by its effects on peripheral or systemic action of insulin on glucose homeostasis, it may do so by a reduction in insulin-related intracellular signaling through the IGF-1 pathway.

It is well documented in human studies that aging is accompanied by slow progressive and irreversible structural changes and functional declines in the heart. Echocardiography in healthy populations from the Framingham Study and Baltimore Longitudinal Study on Aging showed an age-dependent increase in the prevalence of left ventricular hypertrophy, decline in diastolic function, and relatively preserved systolic function at rest but a decline in exercise capacity, as well as an increase in the prevalence of atrial fibrillation (reviewed in [46]). Diastolic heart failure, defined as symptoms of heart failure in the setting of diminished diastolic function, is pervasive in older individuals and markedly increases the risk of mortality [47]. We have previously shown that the mouse recapitulates human cardiac aging by performing non-invasive echocardiographic measurements on WT and mitochondrial-targeted catalase transgenic mice over the course of their lifespans [48]. Echocardiographic parameters were found to show progressive and highly reproducible changes with advancing mouse age. These changes parallel those of human aging, particularly left ventricular mass. We found that old (24 month) male RIIβ^−/−^ mice had attenuated age-related cardiac hypertrophy as well as better myocardial performance indices and Ea/Aa ratios. These data are in agreement with cardiac sparing in adenylyl cyclase (AC5) null mice, which demonstrate an upregulation of both the Raf/MEK/ERK signaling pathway and cell protective molecules such as superoxide dismutase [5]. Like RIIβ, AC is a component of the PKA signaling pathway, and like RIIβ^−/−^ mice, *AC5* null mice are long-lived, suggesting an association of the PKA pathway with cardiac protection and extended lifespan.

Advanced stages of aging are associated with loss of weight in contrast to the increase in weight seen from adolescence through adulthood. Unlike WT mice, RIIβ^−/−^ males, but not females, maintained their weight during their last month of life. Loss of weight culminating in death commonly occurs in mammals at advanced stages and is a hallmark of a geriatric syndrome termed ‘failure to thrive’ [49]. Common diseases associated with failure to thrive are cancer, chronic lung disease, chronic renal insufficiency, liver disease, diabetes, and congestive heart failure [50], and a reduction in any of these age-related diseases may be a contributing factor to the longevity seen in RIIβ^−/−^ males. Another cause of weight loss in advanced age is sarcopenia, or loss of skeletal muscle mass and strength, due to a number of factors including loss of motoneurons, decreased physical activity, altered hormonal status, and decreased food intake [51]–[55]. The lack of weight loss in RIIβ^−/−^ males may be due to an attenuation of sarcopenia, which would represent another aspect of improved healthy aging in these mutants.

The decreased incidence and severity of histopathological lesions in tissues from RIIβ are consistent with the increased lifespan phenotype. However, the small numbers examined histologically at end of life preclude more definitive conclusions about what causes of death were attenuated in the male mice. There were histopathological differences between genotypes that indicated a reduction in age-related kidney dysfunction. Also of interest was the decrease in incidence of lymphosarcoma, which very well may be associated with increased lifespan since this is the most predominant form of cancer in C57BL/6 mice [56]. In contrast to what we see in RIIβ mice, we have reported that long-lived mitochondrially targeted catalase transgenic mice [57] do not suppress lymphosarcoma but do attenuate epithelial cancers [56]. Therefore, different mechanisms are likely involved.

In conclusion, while RIIβ^−/−^ male mice show many indications of attenuated aging, it is still unknown exactly why they live longer. Certainly, the direct association of lifespan with suppressed adiposity is of interest for further study. The genes encoding PKA subunits are conserved from yeast to man, and their disruption has been shown to extend longevity in both yeast [3] and now in mice. Although the yeast TPK genes are PKA catalytic subunit genes, and not regulatory subunit genes like RIIβ, loss of RIIβ in mice causes a concomitant and compensatory decrease in catalytic subunits [7], [8], providing a mechanistic link with loss of function of TPK and extended lifespan in yeast, and loss of function of RIIβ and extended lifespan in mice. That disruption of PKA genes confer extended lifespan in such divergent species suggests a highly conserved role for PKA in longevity, and makes subunits of PKA promising pharmaceutical targets for the treatment of age and obesity-related diseases in humans.

## Materials and Methods

### Animals

The generation and genotyping of RIIβ^−/−^ mutant mice has been described previously [7]. The line is maintained on the C57BL/6 background after more than 10 backcrosses. RIIβ^−/−^ and WT littermate mice used in this study were generated from heterozygote breeding pairs so that age and gender could be equally matched.

### Longevity study

Mice were assigned to cohorts at the time of weaning based on genotype and gender. Mice of both genders on the C57BL/6 background were allowed to live to the end of their natural life. All mice were maintained in the same specific pathogen free housing environment and fed a standard rodent chow ad lib, with no difference in food intake between any of the genotypes. They were housed in gender-specific groups of three to five in individually ventilated cages (Allentown, PA). The housing environment was a specific pathogen free barrier facility, and the room kept at a constant temperature of 22 degrees C, with a 12-hr light/dark cycle. Sentinel animal screening consisted of quarterly testing for infectious bacterial, viral and parasitic pathogens, with all tests being negative throughout the study. The *ad libitum fed* chow (PicoLab Rodent Diet 20) consisted of 20% protein, 5% fat, and 53% carbohydrate. All mice had access to automatically- delivered, reverse osmosis-treated water. Mice were examined daily until their natural death, defined as being found dead in their cage or being unresponsive to touch and euthanized. Kaplan-Meir survival curves were constructed using the known date of birth and date of death for each mouse. The log-rank test was then used to determine statistical differences between cohorts. Maximum lifespan is defined as the mean age of the oldest 20% of mice from each genotype and gender. A total of 20 mice per genotype per gender (80 total) was used to asses survival, with no mice remaining alive at the end of the study. All procedures were performed with the approval of the University of Washington Institutional Animal Care and Use Committee.

### Body weights, serum leptin and QMR

Body weights were measured weekly beginning at 19 to 24 months of age and until end of life. Body weights of an additional cohort of 18 month-old male mice (5 WT and 6 RIIβ^−/−^) were also measured, and their body composition analyzed in vivo using quantitative magnetic resonance (QMR) methods (Echo Medical Systems, Houston, TX). For QMR measurements, conscious rodents were placed in the sample holder and the sample holder then inserted into the center of the magnetic resonance machine. Each animal underwent 3 replicate measurements. The measurement procedure itself causes no harm to the animal. Serum leptin was measured for the following male, WT and RIIβ^−/−^ littermates: 2 month-old (5 WT and 4 RIIβ^−/−^) and 16 month-old (8 WT and 9 RIIβ^−/−^) and the following female WT and RIIβ^−/−^ littermates: 2 month-old (6 WT and 3 RIIβ^−/−^) and 16 month-old (3 heterozygous RIIβ^+/−^ and 2 RIIβ^−/−^). For serum leptin measurements, food was removed from mice 12 h before the collection of blood from the retro-orbital sinus into serum separator tubes (365956; Becton Dickinson, Franklin Lakes, NJ); after separation, plasma was either used immediately or stored at −80°C until analysis. Plasma leptin was measured using an ELISA kit (EZML-82K; LINCO, St. Charles, MO) as per manufacturer's instructions.

### Liver composition

Liver composition analyses were performed on 18 month-old, male mice (5 WT and 6 RIIβ^−/−^ littermates). Mice were euthanized by CO_2_, and livers were dissected out and weighed. A small (approximately 30 mg) piece of the large lobe was set aside for QNMR composition measurements. Composition measurements were measured using quantitative nuclear magnetic resonance imaging (QNMR), with a EchoMRI™ 3-in-1 Animal Tissue Composition Analyzer, at the University of Washington Mouse Metabolic Phenotyping Center (MMPC).

### Blood glucose, serum insulin and insulin sensitivity

Blood glucose was measured on the same day as the initial body weights on all mice from the lifespan cohort at 19 to 24 months of age. For blood glucose measurements, food was removed from mice 6 h before blood was drawn by tail pricking. Analyses were performed using a glucometer and Comfort Curve test strips (Advantage; Accu-Chek, Roche, Basel, Switzerland). Serum insulin was measured for the following male, WT and RIIβ^−/−^ littermates: 2 month-old (5 WT and 4 RIIβ^−/−^) and 16 month-old (8 WT and 9 RIIβ^−/−^) and the following female WT and RIIβ^−/−^ littermates: 2 month-old (6 WT and 3 RIIβ^−/−^) and 16 month-old (3 heterozygous RIIβ^+/−^ and 2 RIIβ^−/−^). For serum insulin measurements, food was removed from mice 12 h before the collection of blood from the retro-orbital sinus into serum separator tubes (365956; Becton Dickinson, Franklin Lakes, NJ); after separation, plasma was either used immediately or stored at −80°C until analysis. Plasma insulin was measured using an ELISA kit (EZRMI-13K; LINCO, St. Charles, MO) as per manufacturer's instructions. Insulin resistance assays were performed on additional cohorts of 6 mice per genotype, for young (2 to 5 month) and old (18 month) males and young (2 to 5 month) females. For insulin resistance assays, mice were first fasted overnight for 16 hours. Mice were then injected intraperitoneally with human insulin (Humulin R; Eli Lilly, Indianapolis, IN) diluted with sterile diluent (Eli Lilly, Indianapolis, IN) at a dose of 1.0 units insulin/kg body wt for the insulin resistance assay. Blood glucose measurements were performed as previously described, at 0, 30, 60 and 90 minutes after injection.

### Echocardiography

Male mice 24 months of age were used for non-invasive echocardiography. Six mice of each genotype (WT and RIIβ^−/−^) were analyzed. The mice were anesthetized by placing them in a flow-through system containing 3–4% isoflurane in a 100% oxygen mix. Following loss of consciousness, the mice were placed on a modified mask assembly that allowed a continuous flow of 1–2% isoflurane in an oxygen mix. The mice could breathe spontaneously, and the depth of anesthesia was monitored by continuous recording of heart rate. Heart rates were not allowed to fall below 400 (near physiological). The mice were taped to an isothermal pad that maintained their body temperature at 37°C. EKG and heart rate were monitored throughout the imaging procedure. Heart rate was determined from a surface electrocardiogram. From a transthoracic approach, two-dimensional targeted M-mode echocardiographic recordings were obtained.

### Histochemistry

End of life (EOL) mice were processed for histochemical analyses. Selected tissues collected at necropsy were fixed in 10% phosphate buffered formalin and processed routinely, sectioned at 4 um and examined by a board-certified veterinary pathologist (PT) blinded to mouse genotype. Morphological diagnoses were assigned for all tissues examined with detailed histopathological evaluation of the kidney and contributing causes of moribundity/death (COD) were assigned as described (56). Renal lesions were scored on a 0–4 severity scale (as described in [56]). Briefly, the renal lesions graded included membranous, proliferative or membranoproliferative glomerulonephropathy, interstitial fibrosis and inflammation, tubular regeneration, degeneration and necrosis, perivascular or peripelvic lymphoid aggregates, pyleitis and mineralization. Non-neoplastic diseases of moderate or severe degree were assigned as contributing to the animal's demise and neoplastic processes were considered if organs were effaced by neoplasia so that function would be altered. Hearts from 18-month-old, (5 WT and 6 RIIβ^−/−^) male mice were also examined as described above. Cardiac lesions were graded on a severity scale from 0–4 and were examined for interstitial fibrosis, cytomegaly, inflammation, mineralization, valvular changes, arteriosclerosis and amyloidosis [56].
