# Correction: Disruption of Protein Kinase A in Mice Enhances Healthy Aging

**DOI:** 10.1371/annotation/d51a16b6-dc82-4cdb-8118-d7c982233d7c

**Published:** 2010-02-05

**Authors:** Linda C. Enns, John F. Morton, Piper R. Treuting, Mary J. Emond, Norman S. Wolf, Dao-Fu Dai, G. S. McKnight, Peter S. Rabinovitch, Warren C. Ladiges

Due to the addition of author Dao-Fu Dai, the author contributions should now read: Conceived and designed the experiments: WL. Performed the experiments: LCE JFM PRT. Analyzed the data: LCE PRT MJE DFD WL. Contributed reagents/materials/analysis tools: NSW GSM PSR WL. Wrote the paper: LCE.

