# Correction: Disruption of Protein Kinase A in Mice Enhances Healthy Aging

**DOI:** 10.1371/annotation/c7cad2dc-1eca-487e-89ae-151a22d8a0b4

**Published:** 2010-02-05

**Authors:** Linda C. Enns, John F. Morton, Piper R. Treuting, Mary J. Emond, Norman S. Wolf, Dao-Fu Dai, G. S. McKnight, Peter S. Rabinovitch, Warren C. Ladiges

An author, Dao-Fu Dai, was omitted from the author byline. The correct author byline is: Linda C. Enns, John F. Morton, Piper R. Treuting, Mary J. Emond, Norman S. Wolf, Dao-Fu Dai, G.S. McKnight, Peter S. Rabinovitch, Warren C. Ladiges.

